# Improved Glycemic Outcomes with Early Initiation of Insulin Pump Therapy in Pediatric Postoperative Total Pancreatectomy with Islet Autotransplantation

**DOI:** 10.3390/jcm10112242

**Published:** 2021-05-21

**Authors:** Siobhan E. Tellez, Lindsey N. Hornung, Joshua D. Courter, Maisam Abu-El-Haija, Jaimie D. Nathan, Sarah A. Lawson, Deborah A. Elder

**Affiliations:** 1 Division of Endocrinology, Department of Pediatrics, Cincinnati Children’s Hospital, Cincinnati, OH 45229, USA; Sarah.Lawson@cchmc.org (S.A.L.); Deborah.Elder@cchmc.org (D.A.E.); 2 Division of Biostatistics and Epidemiology, Cincinnati Children’s Hospital, Cincinnati, OH 45229, USA; Lindsey.Hornung@cchmc.org; 3 Division of Pharmacy, Cincinnati Children’s Hospital, Cincinnati, OH 45229, USA; Joshua.Courter@cchmc.org; 4 Division of Gastroenterology, Cincinnati Children’s Hospital, Cincinnati, OH 45229, USA; Maisam.Haija@cchmc.org; 5 Department of Pediatrics, College of Medicine, University of Cincinnati, Cincinnati, OH 45229, USA; 6 Division of Pediatric General and Thoracic Surgery, Cincinnati Children’s Hospital, Cincinnati, OH 45229, USA; Jaimie.Nathan@cchmc.org; 7 Department of Surgery, College of Medicine, University of Cincinnati, Cincinnati, OH 45229, USA

**Keywords:** pediatric diabetes, TPIAT, islet autotransplantation, insulin pump, diabetes technology

## Abstract

Total pancreatectomy with islet autotransplantation (TPIAT) is a surgical procedure for patients with chronic pancreatitis and poor quality of life. Euglycemia is critical for islet cell survival and engraftment. We reviewed clinical care practice and hypothesized that early in-hospital transition from intravenous insulin to insulin pump therapy, managed by an endocrine unit trained on post-surgical care, would improve glucose control and impact the length of hospital stay. We completed a retrospective analysis of 40 pediatric patients who underwent TPIAT. Comparative hospitalized postoperative groups included those who received insulin intravenously, followed by multiple daily injections, subsequently managed by pump therapy (*n* = 14), versus those who received insulin intravenously followed by early pump therapy provided on the endocrine unit trained to manage post-surgical patients *(n* = 26). The outcomes analyzed included percentage of blood glucoses in target (4.44–6.66 mmol/L (80–120 mg/dL)), hypoglycemia (<3.33 mmol/L (<60 mg/dL)) and hyperglycemia (>7.77 mmol/L (>140 mg/dL)), blood glucose variability, and length of hospital unit stay post-ICU. Hospitalized patients with early transition to pump therapy on a specialized endocrine unit had a higher proportion of glucose values in the target range (61% vs. 51%, *p* = 0.0003), a lower proportion of hyperglycemia (15% vs. 19%, *p* = 0.04), and a lower proportion of hypoglycemia, though not statistically significant (3.4% vs. 4.4%, *p* = 0.33). Early pump users also had lower variability in glucose values over 10 days post-intravenous insulin (*p* = 0.001), and the post-transition median length of stay was shorter by 5 days (median: 11.5 vs. 16.5 days, *p* = 0.005). Early in-hospital pump therapy managed by the specialized endocrine unit improved glucose outcomes and reduced the duration of in-unit stay.

## 1. Introduction

Intense diabetes management, to control both hyperglycemia and hypoglycemia, is required in many areas of medical care. The benefits of maintaining tight glycemic control for insulin-dependent patients in the hospital setting have been well described with regard to patient recovery, decreased length of stay, and patient charge reduction [1]. An emerging population that requires intense insulin management postoperatively is patients with chronic pancreatitis (CP) or acute recurrent pancreatitis choosing surgical treatment with total pancreatectomy with islet cell autotransplantation (TPIAT). The primary goal of the procedure is to alleviate pain and improve the quality of life (pancreatectomy). The secondary goal is to offset the development of diabetes with the potential for patients to become independent from exogenous insulin (islet autotransplantation). Total pancreatectomy by itself results in post-pancreatectomy diabetes, but with islet engraftment and function, both alpha and beta cells allow the patient the potential to secrete endogenous glucagon and insulin, respectively, over time. Because the transplanted beta cells are impaired immediately after transplantation, all post-TPIAT patients require exogenous insulin therapy to quickly create an environment of euglycemia, mitigating the detrimental effect of hyperglycemia on beta cell engraftment and function [2,3,4,5].

Insulin pumps have been extensively studied as an alternative to multiple daily injections (MDI), particularly in pediatric patients for the management of insulin-dependent diabetes [6,7,8,9,10,11]. Insulin pump therapy in type 1 diabetes is associated with improvement in hemoglobin A1C (HbA1C) and reduction in episodes of severe hypoglycemia and diabetic ketoacidosis (DKA) [6,7,9,11]. However, there are limited data on glycemic outcomes in hospitalized pediatric patients immediately following TPIAT. In addition, evidence supporting direct initiation of insulin pump regimens early in the postoperative period, to our knowledge, has not been explored, especially as insulin pumps are rarely initiated at the time of diagnosis for patients with type 1 diabetes.

Current standards recommend maintaining a glucose range of 4.44–7.77 mmol/L (80–140 mg/dL) for the first 3 months after TPIAT [2,12], utilizing MDI to achieve this goal [3,13]. Although there are benefits of using MDI (e.g., most nursing staff in hospitals are certified to administer insulin by subcutaneous injection; injectable insulin is readily available in any hospital setting), this mode of insulin delivery can also present difficulties, such as prolonged dose adjustment intervals, and limitations in dose accuracy in the pediatric population, especially when low incremental dosing is needed. In addition, the half-lives of the long-acting insulins are several days, and multiple adjustments with rapid-acting insulins are required to maintain glucose targets until the long-acting insulin reaches steady-state [10,12,14].

To overcome these barriers, we considered initiating insulin pump therapy in postoperative TPIAT, insulin-naïve pediatric patients while still inpatient. Insulin pump therapy allows for more precise dosing in increments of 0.05 units, while MDI is limited by whole or half-unit administration. The flexibility in adjusting an insulin pump basal rate, which is dosed per hour, allows for more targeted titration of insulin to match time-off enteral feeding schedules. Insulin pump basal delivery can be reduced or suspended completely, which provides both a near-immediate response to hypoglycemia and the ability to address intermittent hypoglycemic trends with targeted titration.

Many hospitals have established guidelines for allowing pump management in insulin-dependent patients, but the patient/caregiver must manage the pump independently during their stay. This is not feasible in a post-pancreatectomy patient as there is no prior experience with pump management. Thus, our study sought to demonstrate the effectiveness of insulin pump therapy in postoperative TPIAT patients, how patients and caregivers can be trained during the postoperative admission, and the effect of the management on glucose targets after operation.

## 2. Materials and Methods

### 2.1. Study Design and Participants

We performed a retrospective analysis of data from two hospital admission clinical care practices at Cincinnati Children’s Hospital for all patients who underwent TPIAT and were previously insulin naïve between 23 April 2015 and 2 March 2019. The study protocol was reviewed and approved by our Institutional Review Board (2019-0608). The data source was collected via retrospective chart review and review of the Pancreas Care Center (PCC) clinical registry.

At our institution, patients in the ICU were managed with IV insulin infusion and utilized a modified insulin titration protocol [12], followed by transfer to an inpatient unit for subcutaneous insulin therapy. Early in the development of the TPIAT program, patients were transferred from the ICU, and insulin therapy delivery was provided by MDI. Glycemic control was achieved using rapid-acting insulin analogs (insulin lispro) in conjunction with a long-acting insulin analog (insulin detemir, dosed every 12 h for continuous basal coverage). After assuring appropriate surgical stabilization, the post-TPIAT patients were then transferred to an insulin pump-trained endocrine unit, where pump therapy replaced MDI. After 2016, there was a change in our in-hospital clinical care practice for post-TPIAT patients. Post-TPIAT patients were transferred directly from the ICU to the endocrine unit where continuous subcutaneous insulin infusion—via an insulin pump—replaced IV insulin. The insulin conversion of total daily dose from IV to subcutaneous pump therapy was equivalent over a 24 h period (compared to the typical increase of 20% when converting from IV insulin to MDI [12]). In order to provide in-hospital pump therapy upon transition from the ICU for a post-surgical patient, all staff nurses on the endocrine unit received insulin infusion pump training and acquired skill competencies in post-surgical management. The population was divided into two groups according to their post-intensive care unit (ICU) clinical care practice. The first group (MDI users) included patients who were managed on MDI prior to in-hospital pump use with transfer to the endocrine unit. The second group (early pump users) included patients who were early in-hospital pump users managed on an endocrine unit throughout their entire post-ICU stay.

Our institution utilizes continuous glucose monitoring (CGM) technology to supplement point-of-care (POC) glucometer testing (the hospital standard) with blood glucose trend information and alerts for impending hypoglycemia. Due to the inaccurate sensor glucose values [15] from hydroxyurea use to control extreme thrombocytosis post-TPIAT [15,16,17], only POC glucometer data were used for analysis. Capillary glucose POC testing was performed every 3 h using an ACCU-CHEK^®^ Inform II Meter (Roche Diagnostics, Basel, Switzerland). This meter is factory calibrated, and hospital policy prompts quality controls (low and high) to be performed every 24 h. The meter package insert (United States) reports an accuracy of 87–98% (±0.28–±0.83 mmol/L; ±5–±15 mg/dL) when reference values are <4.2 mmol/L (<75 mg/dL) and 74.3–99.7% (±0.28–±1.1 mmol/L; ±5–±20 mg/dL) when reference values are ≥4.2 mmol/L (≥75 mg/dL). Regardless of the group, hospital clinical care practice recommended blood glucose monitoring every 3 h or sooner if symptoms were suggestive of hypoglycemia.

All patients were transitioned from the ICU using 24 h continuous enteral feeds via the jejunal port of a gastrojejunal feeding tube. As patients continued to recover, small 2–4 h windows off enteral feeds (time off feeds) were offered.

Data collected included type of pancreatitis (acute recurrent versus chronic), documented genetic panels, age, gender, weight, height, body mass index (BMI), and prior use of insulin. Eventually, all patients were managed with in-hospital insulin pump therapy on an insulin pump-trained endocrine unit and completed comprehensive diabetes education. Dates of admission, operation, transfers, and discharge were additionally collected, and duration of hospitalization was documented. As described, all staff nurses on the endocrine unit received insulin infusion pump training and acquired skill competencies in post-surgical management.

### 2.2. Islet Isolation and Infusion

The islet isolation technique has been described previously [3,5,12,18,19]. Our institution prepared the pancreas in a cold preservation solution and debrided the fat and soft tissue. The pancreas was brought to our local islet isolation facility [5]. A variation of the Ricordi method was used to mechanically and enzymatically digest pancreatic parenchyma and isolate the islets [19]. After processing, the islets were infused through the splenic vein stump into the portal system to take root in the hepatic sinusoids for optimal neovascularization and engraftment [5]. The islets were introduced post-pancreatectomy and while the abdomen was still open [5].

### 2.3. Definition of Glucose Targets

Criteria for defining glycemic outcomes were as follows. Glucose values 4.44–6.66 mmol/L (80–120 mg/dL) were defined as within the target range [1,2,13]. Though many institutions use a target range of 80–140 mg/dL, publications on pediatric post-TPIAT target ranges use 80–120 mg/dL [3,13,19]. Glucose values between 3.33–4.44 mmol/L (60–80 mg/dL) and 6.66–7.77 mmol/L (120–140 mg/dL) were within acceptable range but outside of the target range. For the purposes of reporting results, blood glucose values <3.33 mmol/L (<60 mg/dL) were recorded as hypoglycemic events, and blood glucose values >7.77 mmol/L (>140 mg/dL) were recorded as hyperglycemic events. Glucose values at intervals of at least 180 min apart were used for purposes of analysis. This was to account for the reasonable expected time for rapid-acting insulin to impact the blood glucose trend.

### 2.4. Statistical Analysis

Data were analyzed using SAS^®^, version 9.4 (SAS Institute, Cary, NC, USA). As described above, only POC glucose values were used for analysis in this study. Due to skewed distributions and small numbers, continuous data were summarized as medians with interquartile ranges (IQR: 25th–75th percentiles), while categorical data were summarized as frequency counts and percentages. For continuous data, non-parametric Wilcoxon–Mann–Whitney tests were used to compare characteristics between groups. Chi-square or Fisher’s exact tests were used, as appropriate, for group comparisons of categorical data. Generalized linear models were used to analyze standard deviations over time in order to assess if variability in glucose values decreased the longer the patients were being treated in a unit. A *p*-value <0.05 was considered statistically significant.

## 3. Results

### 3.1. Study Population

In this study, 40 patients underwent TPIAT, of whom 26 were early pump users and 14 were MDI users before transition to in-hospital pump therapy. There was no significant difference in age, sex, BMI percentile, percentage with pancreas-related genetic mutation, or sweat chloride result between the two groups on presentation (Table 1). There was no significant difference in the total islet equivalents per kilogram body weight (IEQ/kg) transplanted between groups (Table 1). One patient in the early pump user group had a positive pre-TPIAT islet autoantibody (insulin autoantibody), and there were more patients with impaired fasting glucose pre-TPIAT in the early pump user group, but neither reached statistical significance (Table 1).

### 3.2. Proportion of Time in Range

**Figure 1:**
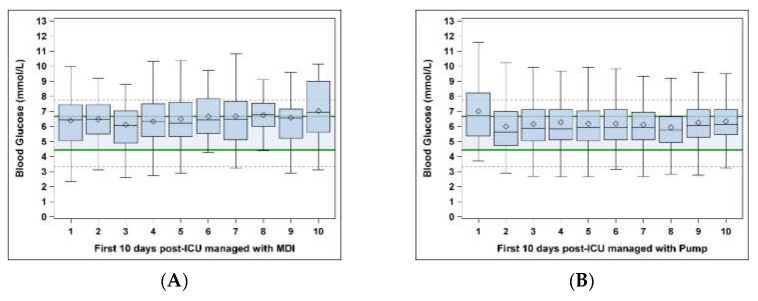
Diamonds represent the mean glucose values; thin lines inside the box plots represent the median glucose values. The shaded box plots represent the interquartile ranges (25th–75th percentiles). The bold horizontal lines mark the target range (4.44–6.66 mmol/L) and the dotted horizontal lines mark the clinically acceptable range (3.33–7.77 mmol/L): (**A**) MDI users; (**B**) pump users.

During the first 10 days post-ICU, a significantly higher proportion of glucose values were found to be in the target range (4.44–6.66 mmol/L (80–120 mg/dL)) in early pump users (61%) compared to MDI users (51%) (Table 2, *p* = 0.0003). The early pump users continued to have a higher proportion of values in a clinically acceptable range (4.44–7.77 mmol/L (60–140 mg/dL)) when compared to the MDI users (82% vs. 77%, respectively; *p* = 0.02). There was a higher proportion, though not statistically significant, of glucose values that were in the hypoglycemic range (<3.33 mmol/L (<60 mg/dL)) for MDI users compared to the early pump users (4% vs. 3%, respectively; *p* = 0.33). There was a significantly higher proportion of glucose values that were in the hyperglycemic range (>7.77 mmol/L (>140 mg/dL)) for MDI users compared to the early pump users (19% vs. 15%, respectively; *p* = 0.04). Figure 1 displays the median glucose values and interquartile ranges for the first 10 days post-ICU for MDI users (Figure 1A) and early pump users (Figure 1B).

### 3.3. Reduction in Variability over Time

**Figure 2:**
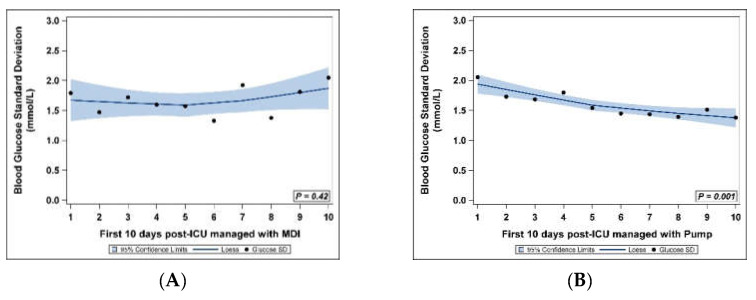
Standard deviation of blood glucoses for the first 10 days post-ICU with 95% confidence limits: (**A**) MDI users; (**B**) pump users.

The variability of the glucose values decreased significantly over the first 10 post-ICU days in early pump users (*p* = 0.001), while the variability did not significantly decrease in MDI users (*p* = 0.42). Figure 2 shows the variability over the first10 days post-ICU in the MDI users (Figure 2A) and in the early pump users (Figure 2B) via loess curve of glucose standard deviation points and the 95% confidence limits.

### 3.4. Length of Hospitalization

Early pump users had a median length of stay on the endocrine unit post-ICU of 11.5 days (IQR: 8.0–15.0). MDI users had a median length of stay on the surgical unit plus endocrine unit post-ICU of 16.5 days (IQR: 14.0–21.0). The length of endocrine unit stay was equivalent between groups, but the early pump user group had a significantly shorter median total length of post-ICU unit stay compared to the MDI group (11.5 vs. 16.5 days, respectively; *p* = 0.005). Incorporating staff competencies on post-surgical management on the endocrine unit permitted the direct transfer to pump therapy. The decrease in patient charge at our institution was an estimated USD 5000 per day by transferring directly to an insulin pump on a pump-trained unit, or UDS 25,000 per hospitalization decrease in patient charges.

## 4. Discussion

Advantages associated with improved glycemic control in hospitalized patients, and the known limitations associated with subcutaneous insulin via MDI, prompted our investigation into the glycemic outcomes for post-TPIAT patients transitioned from IV insulin directly to in-hospital insulin pump therapy. We demonstrated that insulin pump therapy can rapidly achieve target glucose values in postoperative TPIAT patients. This was evidenced by the higher proportion of glucose values in the target range compared to MDI users, fewer hyperglycemic events, fewer hypoglycemic events (though not statistically significant), and lower glucose variability during the first 10 days following discontinuation of IV insulin when compared to MDI users. Additionally, institutional advances in insulin delivery approaches, which included safe insulin pump operation on an inpatient unit as well as specialized nursing training in both endocrine and postoperative surgical care, allowed for successful inpatient insulin pump management, as well as patient/caregiver education on home insulin pump management.

The lower variability in early pump users in the first 24–48 h post-ICU was not related to the creation of enteral feeding windows, as all patients received continuous GJ feeds early in their transition to the hospital units. Alternatively, one might suspect glucose variability to increase with an abrupt stop and start of GJ feeds compared to the continuous delivery of carbohydrate, but this was not the case as windows in the early pump users started day 3–4 post ICU transition and resulted in sustained favorable variability. Given the importance of reducing stress on engrafting islet cells in the immediate postoperative period from even mild hyperglycemia, the greater percentage in the target and the lower variability of glucoses in early pump users are preferred.

While the length of stay on pump therapy was equivalent between groups, investment in training for unit staff to have post-surgical management skills, in addition to insulin pump management skills, allowed direct transition from IV insulin in the ICU to an insulin pump on the endocrine unit. This resulted in a reduction in the overall median length of post-ICU hospitalization at our institution by 5 days, and lower glycemic variability in the first 10 days post-ICU.

Insulin pump outcomes are well understood for insulin-dependent individuals, such as those with type 1 diabetics, but initiation of pump therapy immediately following a diagnosis of insulin-dependent diabetes (such as post-pancreatectomy diabetes) is a novel approach. These findings illustrate benefits that may help explain why typical qualifying criteria required of other diabetes types prior to insurance approval of pump therapy (such as elevated HgA1C, low c-peptide, and evidence of inadequate glucose control) are not applicable for post-TPIAT patients and undermine efforts to optimize the outcomes of islet autotransplantation.

There are several limitations of this analysis. The sample size was small but is a fairly balanced retrospective outcome report of two clinical care practices. Moreover, CGM data were collected during hospitalization but could not be included for analysis due to the effect of hydroxyurea on sensor glucose values, resulting in inaccurate readings. Although the reduction in length of hospitalization is an important finding, there are many factors that affect duration of hospitalization and criteria for determining discharge. Criteria applied when providers consider hospital discharge include the transition from IV to enteral administration of medications, adequate pain management, the ability to maintain hydration and caloric goals without significant nausea and vomiting, and completion of diabetes education. Thus, the shorter post-ICU hospital length of stay in early pump users may not be solely related to the change in clinical care practice and may not be generalizable to all post-TPIAT clinical practices. Lastly, all TPIAT patients at Cincinnati Children’s Hospital used insulin pump therapy and were eventually managed on the insulin-pump-trained unit. We did not have a post-TPIAT group who used only MDI for the duration of the hospitalization following ICU, so we cannot speak of the duration of post-ICU hospitalization when using only MDI.

## 5. Conclusions

Our findings demonstrate that direct transition to in-hospital insulin pump therapy on a specialized endocrine unit with skilled nursing staff results in reduced glycemic variability and provides a higher proportion of glucose values in range during the first 10 days post-ICU. Further studies are needed to assess the impact of early insulin pump initiation on the long-term outcomes of post-TPIAT islet function and insulin independence.

## Figures and Tables

**Table 1:** TPIAT patient characteristics.

	All *N* = 40	MDI Users *N* = 14	Pump Users *N* = 26	*p*-Value *
TPIAT age (years)	13.1 (8.4–15.9)	11.8 (8.9–14.5)	13.4 (7.4–16.5)	0.36
Sex (female)	27 (68%)	10 (71%)	17 (65%)	1.00
Diagnosis				0.54
ARP ^†^	3 (7.5%)	0 (0%)	3 (12%)	
CP ^†^	37 (92.5%)	14 (100%)	23 (88%)	
BMI percentile	70.4 (44.3–93.2)	80.5 (63.2–90.3)	68.3 (42.3–93.4)	0.51
Total islet equivalents/kg (IEQ/kg)	6328 (4298–8346)	6592 (5659–8441)	6166 (3462–7999)	0.26
Genetic testing positive	30 (75%)	13 (93%)	17 (65%)	0.07
* PRSS1*	11/39 (28%)	4/13 (31%)	7/26 (27%)	
* SPINK1*	10/38 (26%)	4/13 (31%)	6/25 (24%)	
* CFTR*	14/38 (37%)	5/13 (38%)	9/25 (36%)	
* CTRC*	0/33 (0%)	0/10 (0%)	0/23 (0%)	
* CPA1*	2/21 (10%)	1/5 (20%)	1/16 (6%)	
More than 1 gene affected	6 (15%)	1 (7%)	5 (19%)	0.40
Islet antibodies (+IAA)	1/38 (3%)	0/12 (0%)	1 (5%)	1.00
Sweat chloride test (CF)				0.58
Negative	25/30 (83%)	9/10 (90%)	16/20 (80%)	
Indeterminate (30–60)	3/30 (10%)	0/10 (0%)	3/20 (15%)	
Positive	2/30 (7%)	1/10 (10%)	1/20 (5%)	
Endocrine insufficiency (impaired fasting glucose/insulin resistance)	4 (10%)	1 (7%)	3 (12%)	1.00
Exocrine insufficiency	17 (43%)	8 (57%)	9 (35%)	0.17

Data presented as median (25th–75th percentile) or n (%). * *p*-value is from testing differences between the MDI and pump user groups. ^†^ ARP (acute recurrent pancreatitis), CP (chronic pancreatitis).

**Table 2:** Proportion of glucose values in target; first 10 days post-ICU.

	MDI Users *N* = 458	Pump Users *N* = 1475	*p*-Value
In target: 4.44–6.66 mmol/L (80–120 mg/dL)	234 (51%)	894 (61%)	0.0003
In clinically acceptable range: 3.33–7.77 mmol/L (60–140 mg/dL)	352 (77%)	1206 (82%)	0.02
Hypoglycemic: <3.33 mmol/L (<60 mg/dL)	20 (4.4%)	50 (3.4%)	0.33
Hyperglycemia: >7.77 mmol/L (>140 mg/dL)	86 (19%)	219 (15%)	0.04
